# Angiotensin-(1–7) exerts a protective action in a rat model of ventilator-induced diaphragmatic dysfunction

**DOI:** 10.1186/s40635-018-0218-x

**Published:** 2019-01-18

**Authors:** Vanessa Zambelli, Anna Sigurtà, Laura Rizzi, Letizia Zucca, Paolo Delvecchio, Elena Bresciani, Antonio Torsello, Giacomo Bellani

**Affiliations:** 1 Department of Medicine, University of Milano-Bicocca, Monza, Italy; 2 Anesthesia and Critical Care, ASST Grande Ospedale Metropolitano Niguarda, Milan, Italy

**Keywords:** Ventilation, Diaphragm, Angiotensin-(1–7)

## Abstract

**Background:**

Ventilator-induced diaphragmatic dysfunction (VIDD) is a common event during mechanical ventilation (MV) leading to rapid muscular atrophy and contractile dysfunction. Recent data show that renin-angiotensin system is involved in diaphragmatic skeletal muscle atrophy after MV. In particular, angiotensin-II can induce marked diaphragm muscle wasting, whereas angiotensin-(1–7) (Ang-(1–7)) could counteract this activity. This study was designed to evaluate the effects of the treatment with Ang-(1–7) in a rat model of VIDD with neuromuscular blocking agent infusion. Moreover, we studied whether the administration of A-779, an antagonist of Ang-(1–7) receptor (Mas), alone or in combination with PD123319, an antagonist of AT2 receptor, could antagonize the effects of Ang-(1–7).

**Methods:**

Sprague-Dawley rats underwent prolonged MV (8 h), while receiving an iv infusion of sterile saline 0.9% (vehicle) or Ang-(1–7) or Ang-(1–7) + A-779 or Ang-(1–7) + A-779 + PD123319. Diaphragms were collected for ex vivo contractility measurement (with electric stimulation), histological analysis, quantitative real-time PCR, and Western blot analysis.

**Results:**

MV resulted in a significant reduction of diaphragmatic contractility in all groups of treatment. Ang-(1–7)-treated rats showed higher muscular fibers cross-sectional area and lower atrogin-1 and myogenin mRNA levels, compared to vehicle treatment. Treatment with the antagonists of Mas and Ang-II receptor 2 (AT2R) caused a significant reduction of muscular contractility and an increase of atrogin-1 and MuRF-1 mRNA levels, not affecting the cross-sectional fiber area and myogenin mRNA levels.

**Conclusions:**

Systemic Ang-(1–7) administration during MV exerts a protective role on the muscular fibers of the diaphragm preserving muscular fibers anatomy, and reducing atrophy. The involvement of Mas and AT2R in the mechanism of action of Ang-(1–7) still remains controversial.

## Background

Mechanical ventilation (MV) is an important tool in the achievement of an optimal pulmonary gas exchange in ICU patients. However, prolonged MV is also associated with numerous potential complications affecting both the lungs and the diaphragm. MV can worsen the injury in previously damaged lung (ventilator-induced lung injury—VILI) [1] and is associated with adverse effects on multiple aspects of diaphragmatic structure and function (ventilator-induced diaphragm dysfunction—VIDD) [2]. The injurious impact of prolonged MV on the diaphragm is tightly related with problems in weaning patients from the ventilator, whose incidence can reach 30% of patients exposed to prolonged MV, with subsequent increase in morbidity and mortality [2, 3]. Several experimental and clinical studies have shown a rapid muscular atrophy and contractile dysfunction in the diaphragm during prolonged MV [4], through the reduction of protein synthesis, the increase of proteolysis, and the activation of oxidative stress. Indeed, animal studies revealed that protein synthesis decreases rapidly already after the first 6 h of MV, and remains low throughout the next 12 h [5]. Simultaneously, the following proteolytic systems initiate: macroautophagy, calpains, caspases, and the ubiquitin-proteasome system [2]. Prolonged MV results in oxidative damage to the diaphragm through the generation of reactive oxygen species and their derivatives that have a significant effect on the contraction of the skeletal muscle [6]. During prolonged MV, the oxidative modification of diaphragm contractile proteins leads to less efficient activation of diaphragm fibers induced by calcium [7].

Renin-angiotensin system (RAS) is a hormonal system implicated in the regulation of blood pressure and fluid and salt balance. Moreover, RAS plays an important role in several pathologies, such as atherosclerosis, myocardial infarction, stroke, diabetes, nephrosclerosis, tumorigenesis, and acute respiratory distress syndrome (ARDS) [8–12]. The classical axis is composed by ACE and angiotensin-II, but, concurrently, RAS has a counter-regulatory one, in which ACE2, the principal peptide angiotensin (1–7) (Ang-(1–7)) and its receptor Mas [13] are involved. RAS could play a relevant role in skeletal muscle diseases, since ACE could act in skeletal muscle, inducing negative effects on myogenesis [14]. The infusion of Ang-II in experimental studies causes marked diaphragm muscle wasting and respiratory muscle dysfunction [15]. Recently, Kwon et al. [16] showed that the treatment with losartan, an Ang-II receptor 1 (AT1R) antagonist, prevented ventilator-induced oxidative stress and diaphragm contractile dysfunction. On the other hand, Ang-(1–7) reduces extracellular matrix proteins, TGF-β levels, and oxidative stress in skeletal muscles [17, 18], thus decreasing fibrosis in muscular dystrophy mouse models. Moreover, Ang-(1–7) and its Mas receptor can maintain muscle strength by preserving fiber diameter, and reduce atrogin-1 and muscle RING-finger protein 1 (MuRF-1) levels during the muscle wasting induced by Ang-II [19]. In line with these results, experimental studies demonstrated that Ang-(1–7) lessens atrophy in models of endotoxin-related skeletal muscle wasting [20] and disuse skeletal muscle atrophy [21]. We conceived the hypothesis that the administration of Ang-(1–7) might have beneficial effects on diaphragm functions by preserving the anatomical structure of muscular fibers [22], in a rat model of VIDD with the use of neuromuscular blocking agent. In this study, we used a rat model, since the human and rat diaphragmatic muscle are anatomically close. Indeed, the composition of fiber types is comparable between the two species [23]. Moreover, we have previously studied the effects of the infusion of Ang-(1–7) in a rat model of VILI [24], showing improvement in terms of oxygenation, inflammation, and lung fibrosis. We also demonstrated that the infusion of Ang-(1–7) is well tolerated by rats during prolonged MV without adverse effects. Ang-(1–7) can bind to the Ang-II receptor 2 (AT2R), even if with lower affinity than Ang-II [25]. Moreover, Mas and AT2R have very similar physiological and pathophysiological actions and are able to form dimers with AT1R, leading to its inhibition [26]. To better characterized the mechanisms of action of Ang-(1–7), we used a Mas receptor (A-779) antagonist and an inhibitor of Ang-II receptor 2 (AT2R) (PD 123319).

## Material and methods

### Animals and husbandry

Sixty Sprague-Dawley rats (250–300 g) were employed (Envigo S.r.l., San Pietro al Natisone, UD, Italy). Animals were housed two per cage in a limited access animal facility, with the following condition: the room temperature was 20 ± 2 °C and the relative humidity set at 55 ± 10%. Artificial lighting provided a 12 h light/12 h dark (7 a.m.–7 p.m.) cycle. The general condition of the animals before the experiment was assessed daily. The care and husbandry of animals were in conformity with the institutional guidelines in compliance with national (D. L.vo 26/2014, Gazzetta Ufficiale della Repubblica Italiana, n.61, March 14th 2014) and international laws and policies (European Union directive 2010/63/UE; Guide for the Care and Use of Laboratory Animals, U.S. National Research Council, 1996). The experimental protocol was approved by the Italian Ministry of Health (531/2016-PR) and by the Animal Care Unit of the University of Milano-Bicocca, Monza, Italy. In full respect of the Reduction principle of the 3Rs, the number of animal/group was selected to obtain reliable results and enough biological samples to perform the analysis planned. Some analysis could not be performed in some animals because of technical problems (e.g., electric stimulator malfunctioning or during histologic procedures); however, the total number of analyses performed in each group is reported in the captions.

### Experimental protocol

Rats were anesthetized with ketamine (100 mg/kg) (Ketavet 100, Intervet Productions, Aprilia, Latina, Italy) and xilazine (4 mg/kg) (Rompun 2%, Bayer, Milano, Italy), orotracheally intubated and ventilated for 8 h (Inspira ASV, Harvard Apparatus, Holliston, MA, USA) with following parameters: tidal volume: 10 ml/kg; respiratory rate: 80/min; PEEP: 2–2.5 cmH_2_O; fraction of inspired oxygen [FiO_2_: 0.5]. Deep anesthesia and paralysis were maintained throughout the whole procedure by infusion in the right femoral artery of propofol (13 mg/kg/h) (Propofol Kabi, Fresenius Kabi Italia, Isola della Scala, Verona, Italy) and ketamine (5 mg/kg/h) and in the right jugular vein of rocuronium bromide (1.5 mg/kg/h) (Rocuronio, Fresenius Kabi Italia, Isola della Scala, Verona, Italy) and ringer acetate (1.8 ml/h). In the left jugular vein, rats received treatment (50 μl/h) depending on the randomly assigned experimental group: (1) sterile saline solution NaCl 0.9% (vehicle), (2) 60 μg/kg/h angiotensin-(1–7) (angiotensin fragment 1–7 acetate salt hydrate, A9202, Sigma Aldrich, St. Louis, MO, USA) (Ang-(1–7)), (3) 60 μg/kg/h angiotensin-(1–7) + 120 μg/kg/h A-779 (A-779 trifluoroacetate salt, SML1370, Sigma Aldrich, St. Louis, MO, USA) (Ang-(1–7) + A-779), and (4) 60 μg/kg/h angiotensin-(1–7) + 120 μg/kg/h A-779 + 120 μg/kg/h PD123319 (PD 123,319 di(trifluoroacetate) salt hydrate, P186, Sigma Aldrich, St. Louis, MO, USA) (Ang-(1–7) + A-779 + PD). Airway pressure and hemodynamic parameters were monitored using pressure transducers in ventilator and arterial catheter, during the whole experimental procedure. A recruitment maneuver (30 cmH_2_O for 10 s) was performed every 60 min, and the plateau pressure and respiratory system static compliance were recorded every hour. A group of unventilated rats was used as control (CTRL).

### Assessment of the injury

The primary outcomes of the study were the anatomical structure of muscular fibers and the levels of atrophy and autophagy. The secondary outcome was to evaluate the effects of Mas and AT2R antagonists.

### Respiratory mechanics

For the lung mechanical properties, a pressure to volume curve was calculated. After a recruitment maneuver, five steps of inspiratory volumes (2.5 ml) were delivered into the lungs. For each step, the plateau pressure was recorded in order to calculate the static compliance.

### Diaphragmatic contractile properties

The diaphragm muscle was excised after animal sacrifice, placed in Krebs solution, and a 2-mm-wide strip was dissected. The strip was mounted into a jacketed tissue bath chamber filled with Krebs solution, containing two stimulation electrodes connected to a stimulator (Grass S88, Grass technologies, Quincy, MA, USA). Tissues were allowed a thermo-equilibration period of 15 min before initiating contractile measurements at 27 °C. The following measurements were made:

- Optimal length (L0) (the muscle length at which the maximal force is recorded): muscle was stimulated at 70 V 100 Hz with 2 ms single pulse and L0 was obtained by systematically adjusting the length of the muscle by using a micrometer while evoking contractions. Thereafter, all contractile measurements were performed at L0
- Peak tetanic tension: the force produced at L0 when diaphragms were stimulated at 70 V 100 Hz with 1000 ms stimulation trains
- Force-frequency relationship: the force-frequency relationship at L0 was determined by sequential 1000 ms stimulation trains of 70 V at different frequency (from 10 to 150 Hz), with 2-min intervals after each stimulation

Muscle force was normalized to tissue cross-sectional area, calculated by the algorithm: [muscle mass/(fiber length × 1.056)], where the muscle mass is the weight of the muscle strip, the fiber length is the L0, and 1.056 g/cm^3^ is the muscle density [27].

### Fiber cross-sectional area and muscle structure

After dissection, right hemidiaphragm tissue was rinsed, embedded in optimal cutting temperature (OCT) and immediately frozen on dry ice. Several 10-μm-thick sections were cut and stained with hematoxylin and eosin. The cross-sectional area (CSA) of the muscular fibers was determined by manually tracing the fiber contour on digitized images. The mean CSA value was calculated on at least 150 fibers per diaphragm. The analysis was performed by two blinded operators.

### Real-time polymerase chain reaction

Total RNA was extracted from a frozen section of diaphragm muscle using Eurogold trifast reagent (Euroclone S.p.A., Pero, Milano, Italy) according to the kit protocol, and quantified using a NanoDrop 1000 spectrophotometer (ThermoScientific, Waltham, MA, USA). Then, 1000 ng of total RNA were incubated with rDNase I (Ambion, Austin, TX, USA) for 20 min at 37 °C to digest contaminating genomic DNA. Further, 400 ng of total digested RNA of each sample were reverse transcribed to cDNA using M-MLV Reverse Transcriptase (Invitrogen, Carlsbad, CA, USA). cDNA was amplified by PCR using GoTaq G2 DNA polymerase (Promega, Milano, Italy) with an Applied Biosystems 7900HT Fast Real-Time PCR System. MuRF-1, atrogin-1, and myogenin were assayed using probe sequences Taqman® Gene Expression Assay (MuRF-1: Fbxo-32 Rn00591730_m1, Atrogin-1: Trim63 Rn00590197_m1, Myogenin Rn01490689_g1, β–actin Rn00667869_m1). Gene expression was measured by the ΔΔCT method and was normalized to β-actin mRNA levels. Data are shown as the fold change of the gene of interest relative to that of control animals.

### Western blot analysis

A section of the diaphragm was immediately frozen in liquid nitrogen and stored at − 80 °C. The cytoplasmic extraction was prepared using an NE-PER Nuclear Cytoplasmic Extraction Reagent kit (Pierce, Rockford, IL, USA) according to the manufacturer’s instruction. Total protein concentrations were quantified by the bicinchoninic acid assay (BCA assay, Pierce, Rockford, IL, USA), and each sample was analyzed according to standard Western blotting protocols. Briefly, 40 μg total proteins of each sample were separated by 4–12% SDS-Page and transferred onto a PVDF membrane (Thermo Fisher Scientific, Rockford, IL, USA). After blocking with 5% skim milk, PVDF membranes were incubated with specific antibody against LC3B (light chain 3, isoform B II, 2775, Cell signaling technology, Danvers, MA, USA) and Tubulin (2125, Cell signaling technology, Danvers, MA, USA) as loading control. After the incubation with horseradish-peroxidase conjugated goat anti-rabbit antibody (7074, Cell signaling technology, Danvers, MA, USA), the final reaction was visualized using enhanced chemiluminescence (Amersham ECL Western Blotting Detection Reagent, GE Healthcare, Buckinghamshire, UK). Images were densitometrically analyzed with ImageJ software (ImageJ 1.50b, National Institutes of Health, USA).

### Statistical analysis

Shapiro-Wilk test was used to assay the population distribution. Comparisons between vehicle and Ang-(1–7) treatment were made by *t* test or Mann-Whitney *U* test, in normally or not-normally distributed data, respectively. Comparisons between Ang-(1–7) treatment and A-779 and PD group were made by a one-way analysis of variance (ANOVA) or by Kruskal-Wallis. If the group effect was significant, a Tukey post-hoc test was used for pairwise comparisons between groups. Data are shown as means ± SD for normally distributed data and as median [interquartile range] when non-normally distributed. In order to calculate the sample size, we started from the study of Kwon et al. [16] and we considered that if we wanted to find a 10% reduction in diaphragm contractile properties with a 80% power and a 0.05 significance level, we had to use ten animals per group. Significance was established at *p* < 0.05 (IBM SPSS Statistics software, version 24.0.0.1).

## Results

### Systemic response

We found no difference in survival in all experimental groups: all rats survived the 8 h of MV, except two rats (one in vehicle and one in Ang-(1–7) group) that were sacrificed after 7 h because of hypotension. The body weight before the experiment and the oxygenation were not different between groups (Table 1). The mean blood pressure was similar between groups at the beginning of the experiment, whereas at the end of the MV it was significantly higher in Ang-(1–7) + A-779 + PD compared to the other two treatment groups (Table 1). Eight hours of MV induced a significant decrease in compliance with no difference between groups.

**Table 1:** Body weight, oxygenation, blood pressure, and compliance during the mechanical ventilation

	Body weight (g)	PaO_2_ (mmHg)	Mean blood pressure (mmHg)		Respiratory system static compliance (ml/cmH_2_O)	
			Start	End	Start	End
CTRL	282 ± 34	–	–	–	0.44 ± 0.04	
Vehicle	279 ± 32	94 ± 15	101 ± 22	91 ± 35	0.41 ± 0.03	0.33 ± 0.05
Ang-(1–7)	275 ± 37	100 ± 6	96 ± 18	91 ± 36	0.41 ± 0.08	0.32 ± 0.05
Ang-(1–7) + A-779	277 ± 30	91 ± 9	99 ± 16	95 ± 43	0.40 ± 0.06	0.32 ± 0.03
Ang-(1–7) + A-779 + PD	276 ± 16	102 ± 11	103 ± 25	139 ± 17*	0.42 ± 0.05	0.34 ± 0.05
ANOVA	NS	NS	NS	0.022	NS	NS

CTRL (*n* = 10): unventilated controls; vehicle (*n* = 18): VIDD + saline treatment; Ang-(1–7) (*n* = 14): VIDD + Ang-(1–7) treatment; Ang-(1–7) + A-779 (*n* = 10): VIDD + Ang-(1–7) + A-779 treatment; Ang-(1–7) + A-779 + PD (*n* = 8): VIDD + Ang-(1–7) + A-779 + PD123319 treatment; *p = 0.018 vs Ang-(1–7), *p* = 0.044 vs Ang-(1–7) + A-779

### Diaphragm contractile dysfunction

**Fig. 1:**
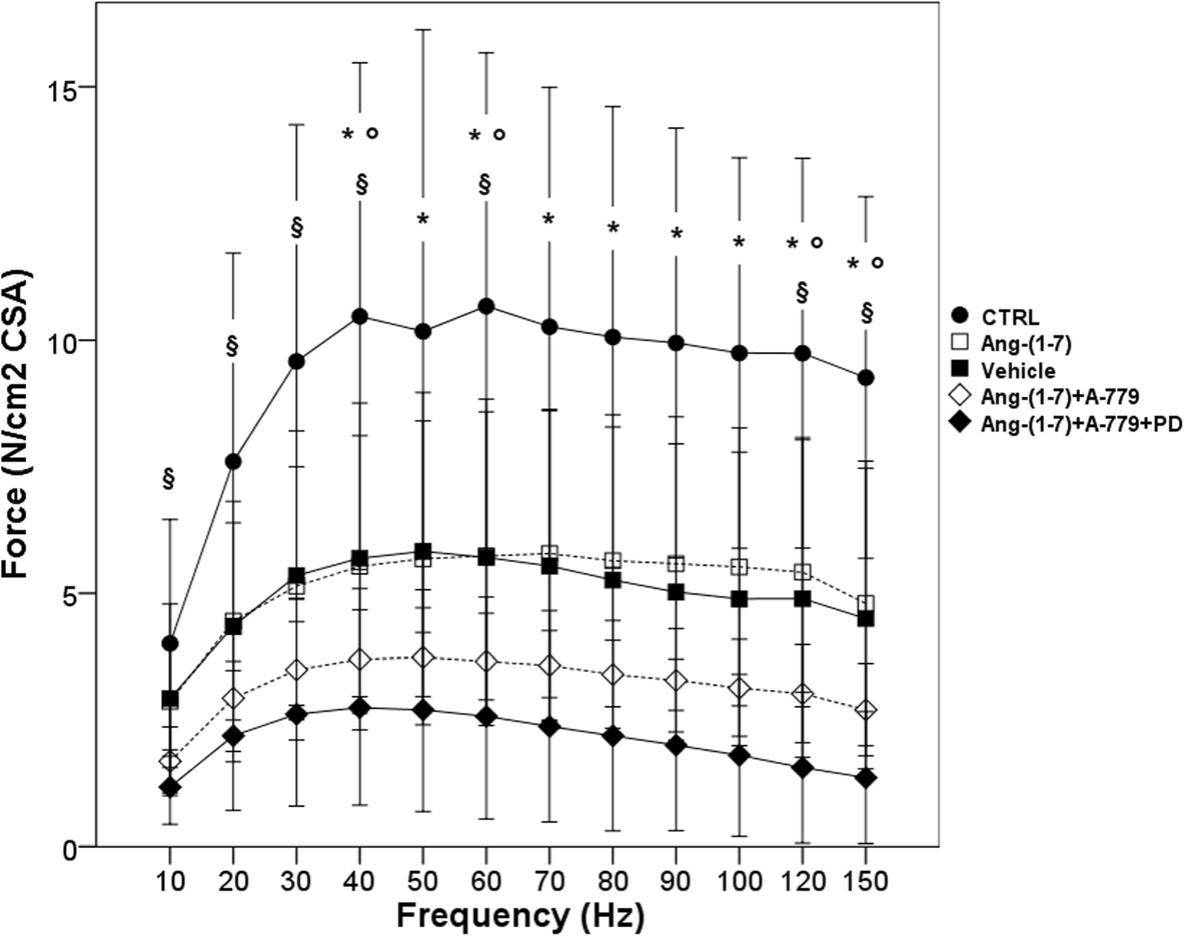
Diaphragm force-frequency relationship. CTRL (*n* = 8): unventilated controls; vehicle (*n* = 10): VIDD + saline treatment; Ang-(1–7) (n = 10): VIDD + Ang-(1–7) treatment; Ang-(1–7) + A-779 (*n* = 10): VIDD + Ang-(1–7) + A-779 treatment; Ang-(1–7) + A-779 + PD (*n* = 7): VIDD + Ang-(1–7) + A-779 + PD123319 treatment; *p < 0.05 CTRL vs vehicle; °*p* < 0.05 CTRL vs Ang-(1–7); §*p* < 0.05 CTRL vs Ang-(1–7) + A-779 and vs Ang-(1–7) + A-779 + PD

After 8 h of MV, all groups showed a significant reduction in diaphragmatic contractility in response to in vitro electric stimulation. As shown in Fig. 1, increasing the frequency of stimulation the diaphragmatic muscle strip of MV rats generated less force than the unventilated (CTRL) diaphragm (*p* < 0.05 for all frequencies and versus all ventilated groups). Ang-(1–7) treatment did not improve the diaphragmatic contraction if compared to vehicle group but, notably, the two groups of rats treated with Mas and AT2R antagonists showed a greater contractility dysfunction, with less force developed at every frequency of stimulation. Indeed, Ang-(1–7) + A-779 and Ang-(1–7) + A-779 + PD groups always showed significantly lower (*p* < 0.05 at 20, 30, 40, and 50 Hz; *p* < 0.01 at the other frequencies) force compared to Ang-(1–7)-treated rats.

### Histological analysis

**Fig. 2:**
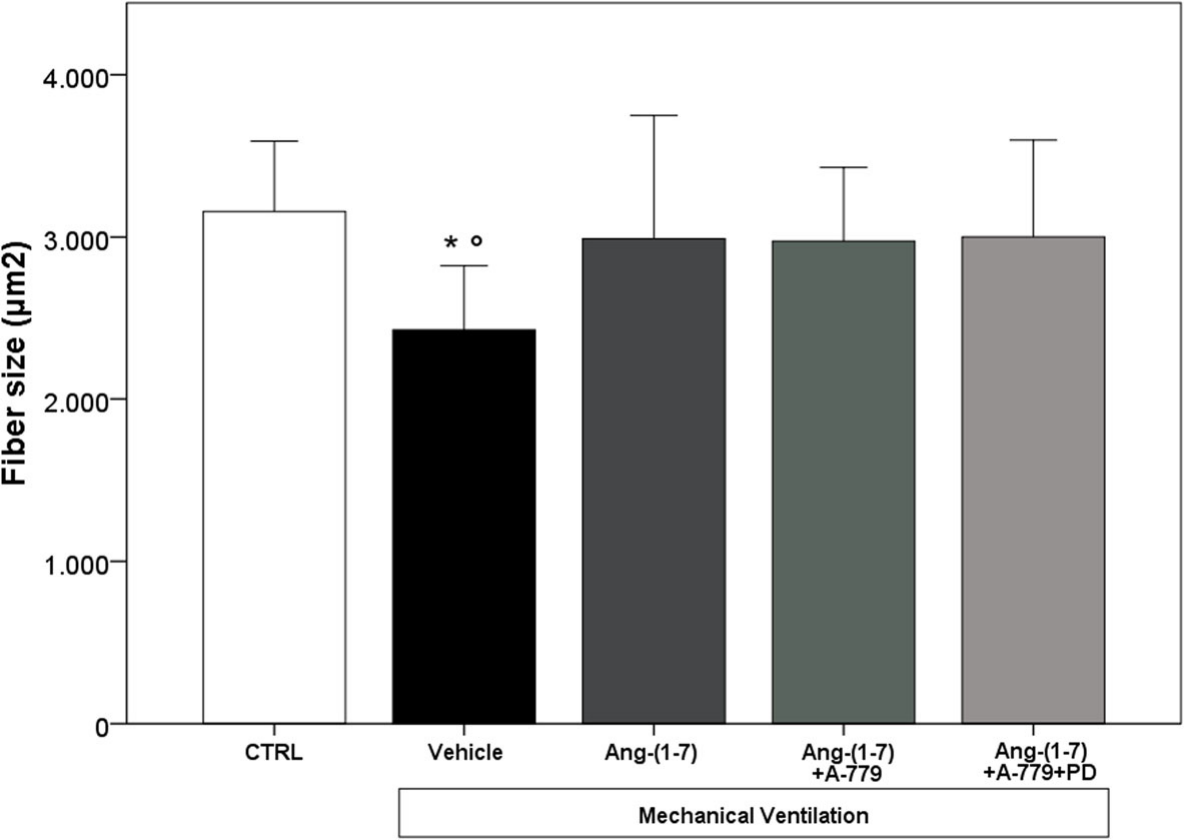
Diaphragm fiber size. CTRL (*n* = 9): unventilated controls; vehicle (*n* = 10): VIDD + saline treatment; Ang-(1–7) (*n* = 13): VIDD + Ang-(1–7) treatment; Ang-(1–7) + A-779 (*n* = 10): VIDD + Ang-(1–7) + A-779 treatment; Ang-(1–7) + A-779 + PD (*n* = 8): VIDD + Ang-(1–7) + A-779 + PD123319 treatment; *p = 0.001 vs CTRL; °*p* = 0.028 vs Ang-(1–7)

Ang-(1–7) administration protected diaphragm muscle fiber from MV-induced cellular atrophy (2990 ± 760 μm^2^). As shown in Fig. 2, in vehicle group, there was a significant decrease (*p* = 0.001) in cross-sectional fiber area (2427 ± 397 μm^2^) if compared to CTRL rats (3159 ± 430 μm^2^). Similar results were obtained in rats treated with Ang-(1–7) + A-779 (2976 ± 455 μm^2^) and Ang-(1–7) + A-779 + PD (3001 ± 595 μm^2^). Interestingly, all Ang-(1–7)-treated rats (with or without antagonists) showed preserved muscular cell structure.

### Real-time PCR

**Fig. 3:**
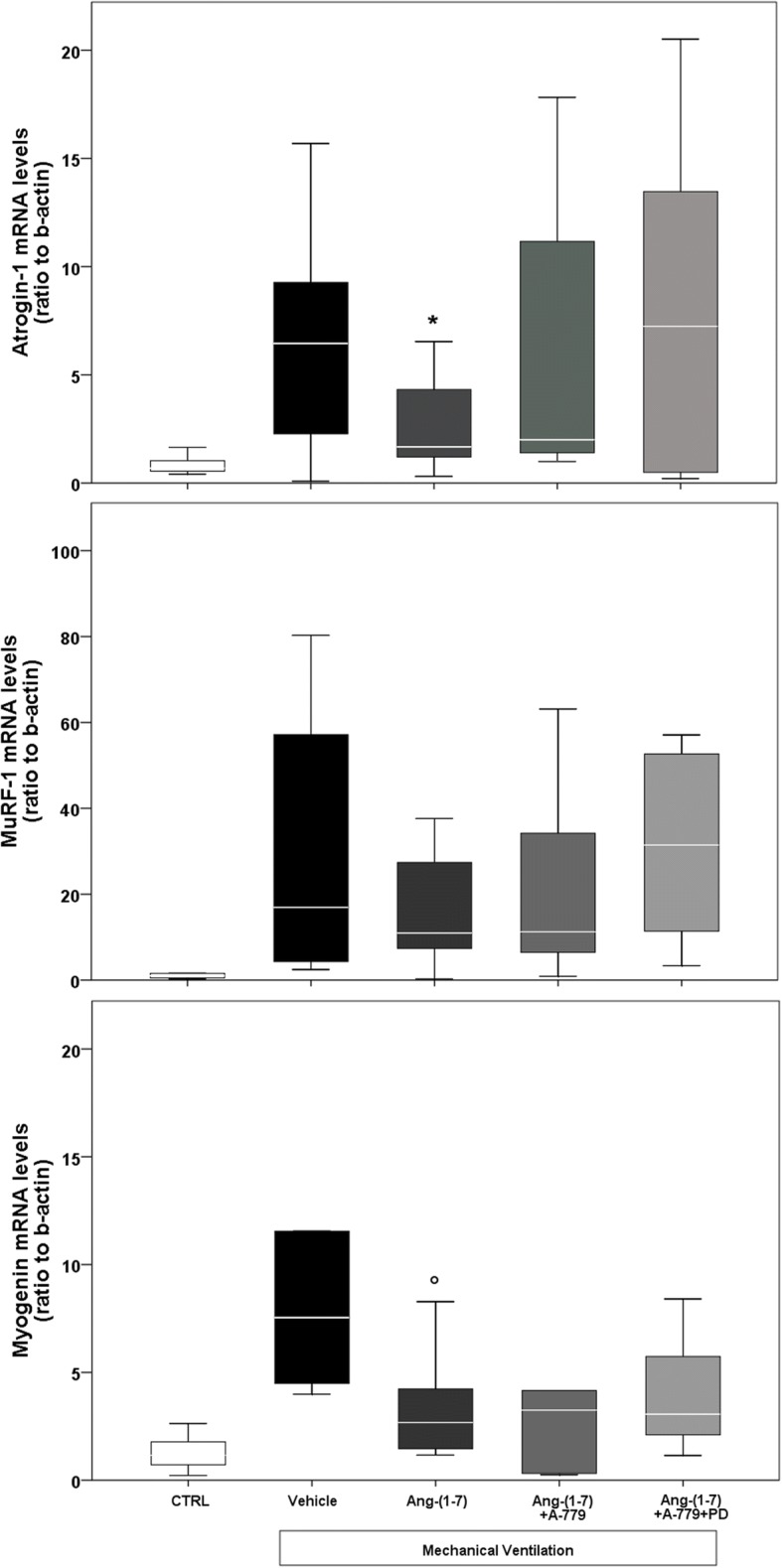
Real-time PCR. CTRL (*n* = 6): unventilated controls; vehicle (*n* = 6): VIDD + saline treatment; Ang-(1–7) (*n* = 6): VIDD + Ang-(1–7) treatment; Ang-(1–7) + A-779 (*n* = 6): VIDD + Ang-(1–7) + A-779 treatment; Ang-(1–7) + A-779 + PD (*n* = 6): VIDD + Ang-(1–7) + A-779 + PD123319 treatment; *p = 0.035 vs vehicle; °*p* = 0.041 vs vehicle

MV induced an increase in mRNA levels of two important muscle-specific E3 ligases (atrogin-1 and MuRF-1) belonging to the ubiquitin-proteasome system of proteolysis (Fig. 3). Interestingly, treatment with angiotensin-(1–7) lowered the expression of these two mRNA (2.82 ± 2.20 and 20.27 ± 23.81 respectively), even if the difference was statistically significant only for atrogin-1 mRNA levels (*p* = 0.035), if compared to vehicle group (6.58 ± 4.62 and 29.43 ± 30.97 respectively). In Ang-(1–7) + A-779 and Ang-(1–7) + A-779 + PD groups, atrogin-1 mRNA levels did not differ from vehicle group, whereas MuRF-1 mRNA levels seemed to have a reduction more pronounced in Ang-(1–7) + A-779 group.

Analogously to CSA results, levels of myogenin mRNA significantly (*p* = 0.041) decreased in Ang-(1–7)-treated rats compared to vehicle group (3.42 ± 2.62 versus 10.25 ± 8.37), and rats treated with antagonists of Mas and AT2R receptor, as already seen in histological analysis, had low myogenin mRNA levels.

### Western blot

**Fig. 4:**
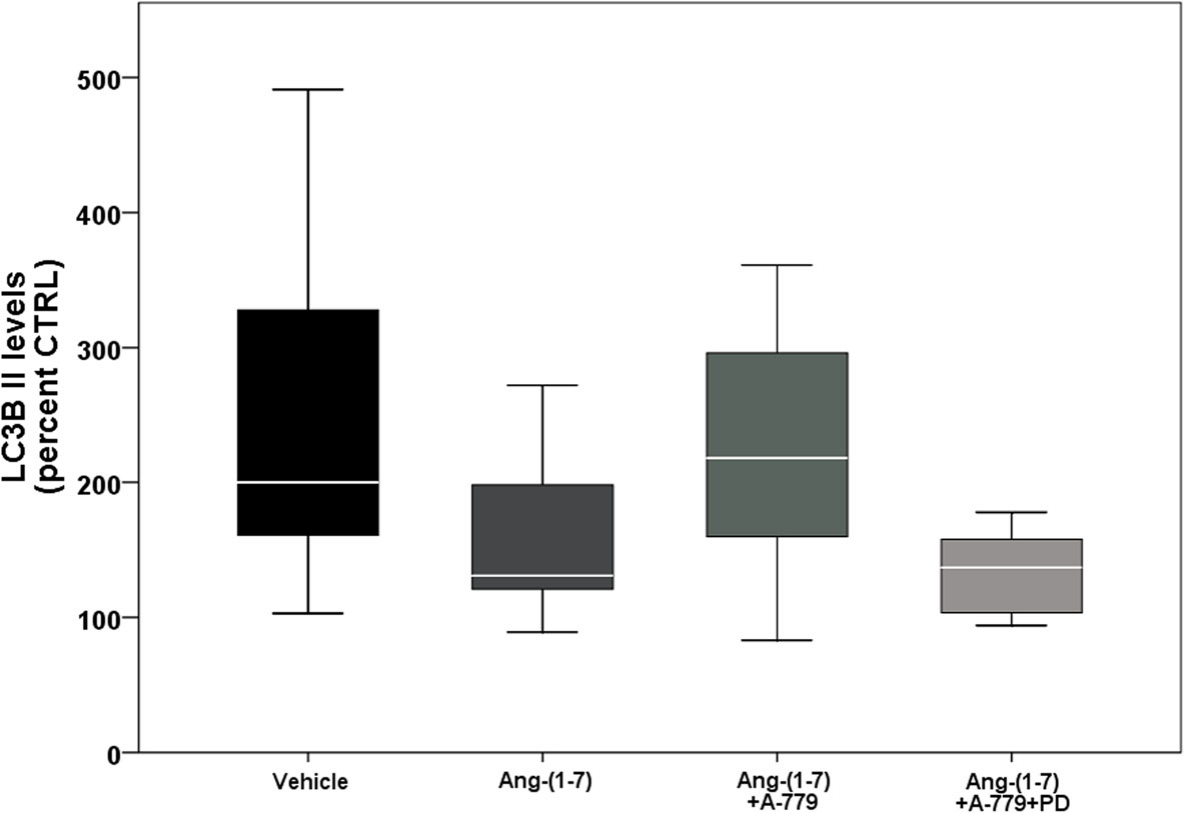
Western blot analysis: autophagy-related protein (LC3B II). Vehicle (*n* = 11): VIDD + saline treatment; Ang-(1–7) (*n* = 10): VIDD + Ang-(1–7) treatment; Ang-(1–7) + A-779 (*n* = 9): VIDD + Ang-(1–7) + A-779 treatment; Ang-(1–7) + A-779 + PD (*n* = 7): VIDD + Ang-(1–7) + A-779 + PD123319 treatment

LC3B II levels were non-significantly (*p* = 0.051) lower in rats treated with angiotensin-(1–7) compared to vehicle group (Fig. 4). Similar levels were found in rats treated with both receptors inhibitor. The group treated with Mas inhibitor had LC3B II levels similar to vehicle.

## Discussion

The RAS is involved in skeletal muscle atrophy, indeed Ang-II acts as key peptide in the regulation of skeletal muscle function, by affecting the tissue structure and muscular contraction in muscle diseases [28, 29]. Conversely, Ang-(1–7) with its receptor Mas [30] can increase muscle strength in dystrophic mice [17] and reduce the muscle atrophy induced by Ang-II. Therefore, the aim of this study was to test the effects of Ang-(1–7) treatment on the diaphragmatic injury induced by the prolonged MV with neuromuscular blocking agent. We have recently [24] demonstrated beneficial effects on the lungs of Ang-(1–7) in a rat model of ARDS. Ang-(1–7) administration during prolonged MV was safe and well tolerated, with no effects on hemodynamics.

### Diaphragm contractile properties

In this study, we decided to ventilate the rats only for 8 h, in order to evaluate the early changes in contractility of the diaphragm and the potential effect of the treatment of Ang-(1–7) on the diaphragm functions. As expected, MV induced a significant reduction in diaphragmatic contractility in response to electric stimulation, as shown in Fig. 1, where the force-frequency curves from all four ventilated groups were shifted toward the bottom of the graph.

### Main effects of angiotensin-(1–7) treatment

From the functional standpoint, Ang-(1–7) did not induce an improvement of the contractility force. The main finding of this study is the protective role of Ang-(1–7) treatment on the muscular fibers of the diaphragm: the CSA and the myogenin mRNA levels were significantly higher and lower, respectively, than in vehicle group. The reduced CSA is an index of diaphragmatic atrophy that occurs also in clinical settings in the first hours of MV. Myogenin is a muscle regulatory factor, whose levels are pronounced after MV [31], and is implicated as modulator of fiber phenotype [32]. Myogenin mRNA is mainly found in slow-twitch muscle [33], so its high levels are correlated to fibers switching from fast to slow [32]. Since the balance between protein degradation and protein synthesis regulates skeletal muscle fiber size, the reduction in muscular atrophy may depend on the levels of atrogin-1 and MuRF-1. Eight hours of MV induced an increase of their mRNA levels, as shown in Fig. 3, but they were reduced in Ang-(1–7) group. Atrogin-1 and MuRF-1 are specific E3 ligases, belonging to ubiquitin-proteasome system, that play an important role in muscle protein degradation and in skeletal muscle atrophy [34, 35]. Numerous studies in humans and animals demonstrated that MV stimulated the expression of E3 ligases along with an increase in ubiquitinated proteins [36–38]. In order to get a basic estimate of autophagy activity, we measured the protein levels of LC3B II, a microtubule-associated protein. Autophagy is an important cellular process that involves sequestration of proteins and cell organelles in autophagosomes and degradation in lysosomes, which is activated in response to a variety of stress-related diseases [39]. In our study, LC3B II levels tended (*p* = 0.051) to be reduced in Ang-(1–7) group compared to vehicle, showing an anti-autophagic effect.

Unfortunately, the preservation of fiber structure and the decreased protein degradation was not associated to an improvement in force generation, as compared to vehicle treatment. This finding questions the potential clinical relevance of Ang-(1–7) but deserves further scrutiny, since it might be related to the timing of our analysis. It is possible that if MV would have been prolonged beyond the 8 h, the vehicle group would show a further decrease in contractility caused by the atrophy of muscle fibers, prevented by Ang-(1–7).

### Use of antagonists of Ang-(1–7) receptors

The inhibition of the two receptors Mas and AT2R affected in part the diaphragm contractility function: both treatments led to significant lower force developed in comparison to Ang-(1–7)-treated rats. Interestingly, also Mas (A-779) and AT2R (PD123319) receptor antagonists preserved diaphragm fibers in a way similar to Ang-(1–7). As expected, LC3B II levels remained high with A-779 treatment, whereas in the group treated with PD123319 levels were similar to those of Ang-(1–7)-treated rats. This is in contrast with a study of Jiang et al. [40] that demonstrated that both A-779 and PD123319 were capable to revert the anti-autophagic effect of Ang-(1–7).

The controversial results obtained in the rats treated with the antagonists of Mas and AT2R did not clarify the mechanism of action of Ang-(1–7) and they are in line with Villela et al. [26] that described the unclear nature of the interaction between these two receptors. At the hemodynamic level, we found that, as expected from our previous study [24], no difference was evident between CTRL, vehicle, and Ang-(1–7) groups, whereas the treatment with PD123319 induced a significant increase of blood pressure. From the functional point of view, the diaphragm in A-779 and A-779 + PD123319 groups were characterized by a significant reduction in contractile capability, demonstrating an important role of Ang-(1–7) for the contraction. This aspect could suggest that the RAS shifts the action on the “dark side” of the system, composed by Ang-II and AT1R, with negative effects. Histological analysis and quantification of myogenin mRNA indicated that muscle fibers are protected from atrophy in all three groups treated with Ang-(1–7) with or without antagonists. It is possible that in the presence of the two blocked receptors, Ang-(1–7) circulating levels increased and its alternative metabolites, such as alamandine, are synthesized and can act through different receptors [41]. Indeed, alamandine can be produced directly from Ang-(1–7) through decarboxylation of N-terminal aspartate amino acid residue [42]. These two peptides have high similarity and the biological actions seem to be closely related to each other, although each compound acts through different receptors [41].

### Experimental limitations

This study has some limitations which should be acknowledged. First, during the MV period, the rats received an infusion of rocuronium bromide that could probably affect the diaphragmatic muscle. Indeed, it is known that, concurrently to mechanical ventilation, some drugs, such as neuromuscular blocking agents, may worsen diaphragm dysfunction, albeit data on their effects are not univocal [43–46]. Second, we obtained quite low values of force during in vitro electric stimulation if compared to other studies in literature [5, 47–49]. This could be due to the relatively young age of rats used in the present study: we used 8-week-old rats. The age-related changes in contractile properties of diaphragm are known [50], and it could have influenced our measures. Third, we did not distinguish the different type of muscle fiber in the histological analysis, but for the measure of the cross-sectional area, we used hematoxylin and eosin staining instead of immunohistochemistry. Fourth, we anesthetized the animals with propofol and ketamine even if it was demonstrated that these anesthetics could affect per se the diaphragm contractility and autophagy [51–53]. Fifth, we did not include a group or rats treated with losartan (AT1R blocker), that was already demonstrated to have beneficial effect on VIDD [16]. Finally, we did not evaluate the effects of Ang-(1–7) on the oxidative stress.

## Conclusions

To our knowledge, no studies investigated the role of Ang-(1–7) on the diaphragmatic dysfunction. Our results show that Ang-(1–7) preserved the muscle fibers from atrophy, probably by reducing the expression of the two major E3 ligases, atrogin-1 and MuRF-1 as confirmed by the lower levels of myogenin mRNA, typically expressed by slow fibers. These beneficial effects on fiber structure, however, were not mirrored by an increased force generation capability. These results encourage further studies preliminary to the use of angiotensin-(1–7) in humans.

## Abbreviations

Ang-(1–7): Angiotensin-(1–7)
ARDS: Acute respiratory distress syndrome
AT1R: Ang-II receptor 1
AT2R: Ang-II receptor 2
CSA: Cross-sectional area
MV: Mechanical ventilation
RAS: Renin-angiotensin system
VIDD: Ventilator-induced diaphragmatic dysfunction
VILI: Ventilator-induced lung injury
